# Wrapping grafting for congenital pseudarthrosis of the tibia: A preliminary report

**DOI:** 10.1097/MD.0000000000008835

**Published:** 2017-12-01

**Authors:** An Yan, Hai-Bo Mei, Kun Liu, Jiang-Yan Wu, Jin Tang, Guang-Hui Zhu, Wei-Hua Ye

**Affiliations:** Department of Pediatric Orthopaedics, Hunan Children's Hospital, The Pediatric Academy of University of South China, Changsha, Hunan Province, P.R. China.

**Keywords:** autogenic iliac bone, congenital, enclosing grafting, external fixator, intramedullary rod, pseudoarthrosis of tibia

## Abstract

**Objective:**

Treatment of congenital pseudarthrosis of the tibia (CPT) remains a challenge. The autogenic iliac bone graft is important consistent of treatment for CPT. The purpose of this study was to investigate the role of wrapping autogenic iliac bone graft in improvement of the curing opportunities of CPT.

**Methods:**

We combined Ilizarov fixator with intramedullary rodding of the tibia and wrapping autogenic iliac bone graft for treatment 51 cases of CPT between 2007 and 2010. The mean age is 3.2 years at index operation, of which 31 patients (61%) were below 3 years old. According to Crawford classification, 5 tibia had type-II morphology; 3, type-III; 43, type-IV.

**Results:**

In the postoperative follow-up of 3.5 months (range from 3 to 4.5 months), all cases were found that the bone graft sites of pseudarthrosis of the tibia showed a significant augmentation and spindle-shaped expansion as obvious change. All cases of this series have been followed-up, average followed-up time were 1.6 years (range from 7 to 3.1 years), of which 19 cases were more than 2 years. The average time of removed the Ilizarov ring fixator was 3.5 months (range from 3 to 4.5 months). According to Johnston Clinical evaluation system, 26 cases had grade I, 21 cases, grade II, 4 cases, grade III. Following the Ohnishi X-ray evaluation criteria, union of pseudarthrosis of the tibia were 42 cases, delayed union 5 cases, nonunion 4 cases.

**Conclusion:**

Autogenic iliac bone graft is able to offer the activity of osteoblasts and osteogenesis induced by bone morphogenetic protein (BMP) and glycoprotein, meanwhile enclosing bone graft could help keep cancellous bone fragments in close contact around pseudarthrosis of the tibia, allowing the formation of high concentration of glycoprotein and BMP induced by chemical factors because of established the sealing environment in location, all of which could enhance the healing of pseudarthrosis of the tibia.

## 1 Introduction

The surgical approach to the treatment of congenital pseudarthrosis of the tibia (CPT) include vascularized bone grafting, intramedullary stabilization, and external fixation, of which have difference in achievement of union, as well as complication such as refracture, valgus angulations, and leg-length discrepancy.^[1–4]^ However, the therapy effects of the combined surgical techniques in treatment of CPT were not satisfactory due to the unavoidable results of amputation and severe disability.^[5]^ We have developed a combinative surgical technique that includes Ilizarov's fixator with intramedullary rodding of the tibia and wrapping autogenic iliac bone graft for the treatment 51 cases of CPT in children with a high rate of union between February 2007 and March 2010. The purpose of this study was to describe the technique of wrapping autogenic iliac bone graft and evaluate the short-term outcome of this combinative technique.

## 2 Materials and methods

From February 2007 to March 2010, 51 patients with CPT were treated with Ilizarov's fixator, intramedullary nailing of the tibia and wrapping autogenic iliac bone graft in our department. The mean age at the index operation was 3.2 years (range, 10 months–12.5 years). In which there were 10 girls and 41 boys. According to Crawford classification,^[6]^ 5 tibia had type-II morphology; 3, type-III; 43, type-IV. Among these patients, 38 patients (74%) had no previous surgery, 11 (26%) had undergone more than 1 unsuccessful procedures (range, 1–4 procedures). The nonunion was always located on the distal third of the leg. Forty-five patients (88.2%) presented with neurofibromatosis type 1. The etiology was 45 patients with neurofibromatosis-1 (88.2%), and 6 with unknown etiology (23.3%). In 27 patients the right side was affected (52.9%), the left was affected in the other 24 (47.1%). The initial limb-length discrepancy (LLD) was measured and the mean preoperative LLD was 3.8 cm (range, 0–7 cm). The study was approved by the Ethics Committee of Hunan Children's Hospital, The Pediatric Academy of University of South China and all patients signed the informed consent.

### 2.1 Surgical technique

#### 2.1.1 Harvesting autogenic iliac bone

**Figure 1:**
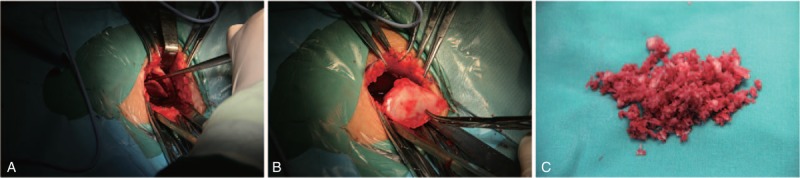
Exposure of outer table of ilium (A), harvesting a square of cortex sized 4 cm × 4 cm (B) and lots of cancellous bone curetted from supra-acetabular region (C).

**Figure 2:**
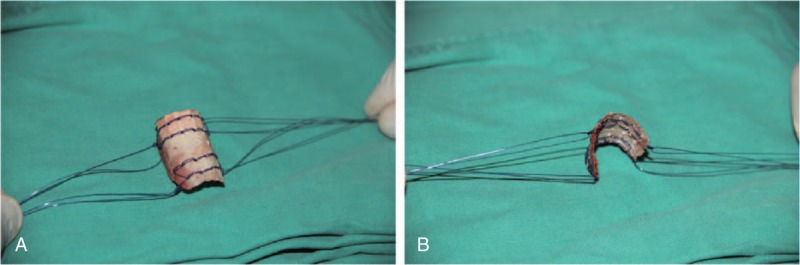
The square of cortex was made lots of holes with Kirschner wire and sutured with absorbable sutures (A), and note the square of cortex with cylindrical shape in which there were double longer sutures on each corner for wrapping cancellous bone graft (B).

The patient was positioned supine on an operating table with a pillow under the ipsilateral buttock. The anterior iliac crest bone graft is first harvested through a straight incision centered over the anterior superior iliac spine. The apophysis was split and subperiosteal exposure of the outer table of the anteriolateral surface of the ilium. At first the cortex sized 4 cm × 4 cm was obtained from the outer wall of the ilium and as much cancellous bone as possible curetted from supra-acetabular region, but keeping inter wall intact (Fig. 1). After those manipulations, many holes were made in the squared cortex with fine Kirschner wire and the squared cortex was weaved with absorbable sutures in order to mold cylindrical shape for wrapping graft (Fig. 2).

#### 2.1.2 Excision of pseudarthrosis with fixation of intramedullary rodding and Ilizarov fixator

The tibia was approached through an anterior incision over the site of the pseudarthrosis and just lateral to the tibial crest. The deep fascia of the anterior compartment was divided at this level. The fibrous Hamartoma and abnormal bone surrounding the site of the pseudarthrosis were then excised completely to the normal shaft of the tibia. The medullary canal of both tibial fragments was reamed with a drill until suitable rod could be inserted. If the fibula had the pseudarthrosis, the fibrous tissue at site of fibula also needed excision. In case of intact fibula which could prevent the apposition of the tibial fragments, it should be osteotomized and fixed with an appropriately sized Kirschner wire after intramedullary rodding of the tibia.

The William rod was then inserted into medullary canal of the tibia across the CPT site performed from distal to proximal via talus and calcaneus and out through the heed pad. IM rods were consists of an indwelling and an insertion rod that was coupled together. It is necessary that the insertion rod was in center of distal tibial physis under supervision of the C-arm in order to keep natural dorsiflexion–plantar flexion of the foot and natural varus-valgus of the ankle. Then the rod was driven retrograde into proximal tibial fragment which is anatomically aligned in both the coronal and the sagittal planes and was verified using intraoperative imaging.

After finishing the fixation of intramedullary rod, the Ilizarov's fixator was installed with one whole ring above the nonunion site and one below. Of which either ring was fixed with 2 or 3 1.8 mm tensioned Kirschner wires through the tibia and the 2 Ilizarov's ring was connected together by 4 threaded rod and applying appropriate pressure at site of pseudarthrosis. If distal tibial segment was less than 3 cm in length, a calcaneal half-ring or U-sharp ring was used to increase distal stability. In addition, concurrent segmental bone transportation was performed in the 8 cases, because the tibial discrepancy was more than 5 cm with normal bone of proximal tibia.

#### 2.1.3 Wrapping autogenic iliac bone graft

**Figure 3:**
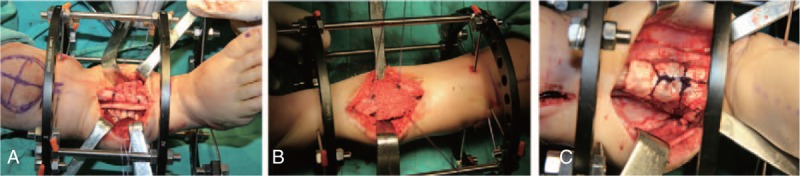
The cylindrical cortex wrapped pseudoarthrosis of the tibia (A), a lots of cancellous bone bad been compacted circumferentially between the cortex and the pseudarthrosis site (B), and the wrapping bong graft finished by tied the sutures (C).

The cylindrical shape cortex was then wrapped around the bone ends of the pseudarthrosis site following application of the Ilizarov fixator. The cancellous bone graft was placed circumferentially between the cortex grafted and the pseudarthrosis site and tied the absorbable sutures which had been connected to the cortex on each corner in order to establish the sealing environment in pseudarthrosis site for enhancement of induced osteogenesis (Fig. 3).

In addition, 8 of the 15 patient had a concomitant pseudarthrosis of the fibulae. We tried to fix these fibulae with a thin intramedullary Kirschner wire.

#### 2.1.4 After treatment

Before closure of wound the deep fascia of anterior compartment of the leg was incised to prevent compartment syndrome. Thereafter the wound was routinely sutures in layers over a drain. The drain was routinely removed and nonweight-bearing anteroposterior and lateral radiographs of the involved extremity were made at 6 and 12 weeks respectively following index operation. When the pseudarthrosis of the tibia has been consolidated, the Ilizarov's fixator was removed and a short leg cast was applied for 2.8 months (rang from 2 to 3.5 months). A protective knee-ankle-foot orthosis was then used with weight-bearing walking until skeletal maturity. Complications such as infection, drainage, and wound dehiscence were recorded.

#### 2.1.5 Clinical and radiographic evaluation

According to Johnston clinical graded system,^[3]^ the outcome was evaluated at the time of the recent follow-up, in which grade 1 indicates unequivocal union; grade 2, an equivocal union (a residual transverse or longitudinal cortical deficiency) and/or deformity (usually >15° of valgus, procurvatum, or recurvatum); and grade 3, a persistent nonunion or refracture.

Degree of union on radiographs was evaluated using the criteria established by Ohnishi et al,^[4]^ which included 3 grades. Radiographic union was defined as possessing continuity of bone density between the fragments without obvious radiolucent zone between them and possessing cortex-bridging fragments with sufficient thickness and radiodensity on both anteroposterior and lateral radiographs. Delayed union was defined as a process of healing that was slow but was progressing. Nonunion was defined by the healing process that had completely ceased.

## 3 Results

The same surgical technique was used in 51 patients with CPT and all of patients who were fully follow-up and the average follow-up was 1.6 years (range from 6 months to 3 years 1 month), of which 19 patients were follow-up more than 2 years. The cortex thickens and spindle augmentations at the site of wrapping autogenic iliac bone graft were observed on radiographs in total 51 patients at time of 3 months. The duration of the Ilizarov fixator treatment was an average of 3.5 months (range from 3–4.5 months) (Table 1).

**Table 1:**
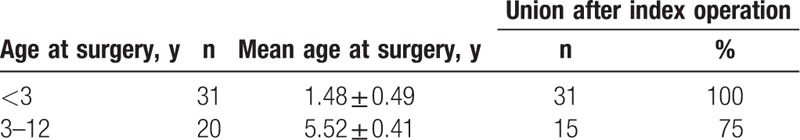
Frequency of primary union of pseudarthrosis of the tibia after the index operation.

According to Johnston clinical graded system,^[3]^ 26 patients (50.9%) had a grade-1; 21 patients (41.1%), grade-2, and 4 patients (7%), grade-3 outcome.

**Figure 4:**
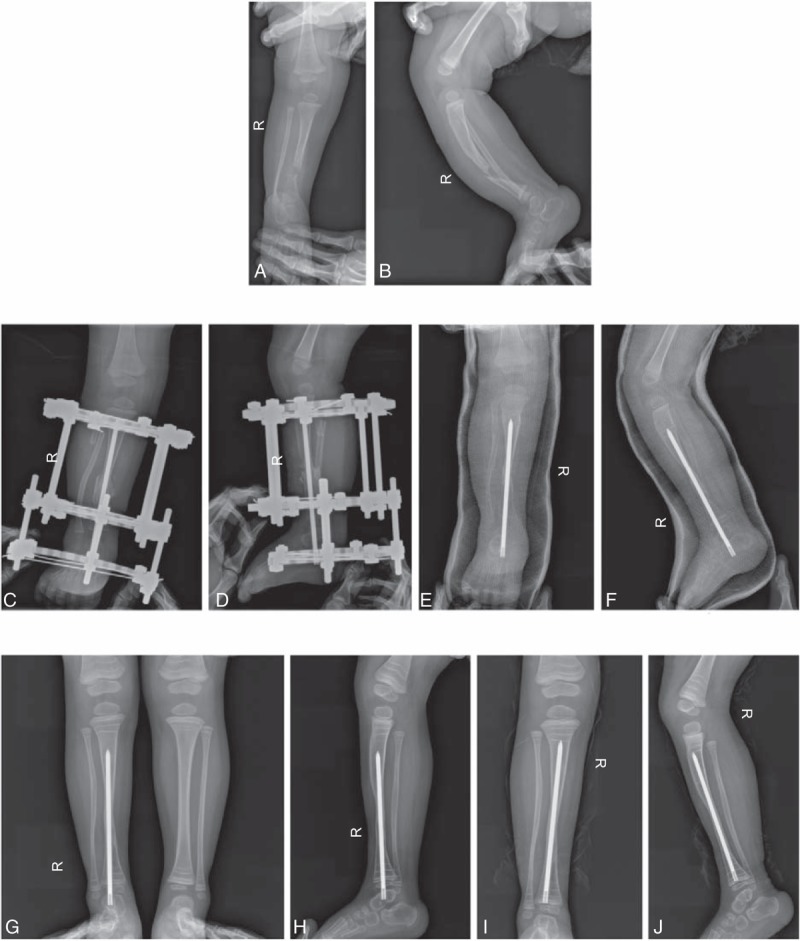
Typical case: Preoperative frontal (A) and lateral (B) radiographs of a 2-year-old boy, showing congenital pseudarthrosis of the right tibia and fibulae with neurofibromatosis type 1. Before removing of Ilizarov apparatus no any gaps existed between wrapped graft and previous pseudarthrosis of the tibia on frontal (C) and lateral (D) radiographs at 3 months after operation. Anteroposterior (E) and lateral (F) radiographs showed union of previous pseudarthrosis with evident spindle-shaped expansion in site of wrapping graft at 6 months after operation. Anteroposterior (G) and lateral (H) radiographs exhibited some remodeling of previous pseudarthrosis with reduced expansion in site of wrapping graft at 15 months after operation. Anteroposterior (I) and lateral (J) radiographs revealed continuous remodeling of previous pseudarthrosis with apparent increase of thickness and density of cortex in site of wrapping graft at 3 years following operation. The distal tip of rod had been pushed into distal epiphysis of the tibia because of precluding functional motion of the ankle joint.

Based on radiographic criteria by Ohnishi et al,^[4]^ union of the tibial pseudarthrosis was achieved in 32 of 51 cases with a mean time to union of 6.1 months (range, 5–8 months). There were 15 patients in the state of delayed union and 4 patients left ununited (Table 2, Fig. 4).

**Table 2:**
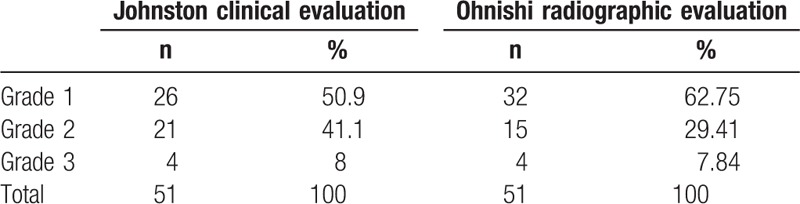
Clinical and radiographic evaluation after the index operation.

### 3.1 Complications

There were no operatively complications, such as neurovascular damage or compartment syndrome. Pin-tract infection occurred in 6 of the 51 patients successfully treated by local care. But the 10 patients without simultaneous lengthening had a mean lower-extremity length discrepancy of 2.8 cm (range, 0.5–4.5 cm). Forty patients of total 51 case had sequels of remarkable ankle valgus, of which there were 3 patients in station I, 21 cases, in station II, and 16 patients in stations III by Malhotra classification of ankle valgus.^[7]^

## 4 Discussion

Regardless of the ongoing developments of new approaches or the improvement of the current ones for the treatment of CPT, their management continues to be difficult, even for the more experienced orthopedic surgeons.^[8–10]^ It is well known that the autogenic bone graft played an important role in treatment of CPT, but the mechanism of union of CPT have been poorly understanding following autogenic bone graft.^[11]^ Many clinical researches have demonstrated that the mechanical stabilization and biologic stimulation remains the gold standard treatment for most nonunion after fractures.^[2,8,12–15]^ Because of the pathologic fracture, unfortunately, CPT of treatment is the most difficult than the nonunion after fractures. Autogenic bone grafting could provide a unique biologic function promoting union by stimulating the local biology at the nonunion site.^[16]^ The biologic mechanisms that provide a rationale for bone grafting are osteoconduction, osteoinduction, and osteogenesis.^[17]^ Osteoconduction is a property of a matrix that supports the attachment of bone-forming cells for subsequent bone formation.^[18]^ Osteoinduction involves the stimulation of osteoprogenitor cells to differentiate into osteoblasts that then begin new bone formation.^[19]^ The most widely studied type of osteoinductive cell mediators are bone morphogenetic proteins (BMPs), but osteogenesis is a relatively new term that can be defined as the generation of bone from bone-forming cells, along with bone growth generated via the other 2 mechanisms.^[20,21]^

Although bong graft substitute and xenograft possess function of osteoinduction as well as osteoconduction.^[22,23]^ Autogenic cortex and cancellous grafting remains the gold standard.^[24]^ It is necessary that both of recipient and bone grafted have close contact over a large surface area along with good blood supply for bone graft, the autogenic cortex and cancellous grafting could successfully incorporate into recipient site. As native bone grows, it will generally replace the graft material completely resulting in a fully integrated region of new bone.

Although some authors attempted to use rhBMP-7 or biomembrane for treatment of CPT, of which also have affects with either osteoinductive or both osteoconductive and osteoinductive properties, the results were disappointment.^[25]^ Therefore most authors advocate autogenic bone grafting or combined with BMP to treat refractory congenital pseudarthrosis of tibia.^[26,27]^ We developed a procedure of wrapping bone graft combined autogenic cortex and cancellous bone, which can completely satisfy biological functions of bone graft. Even though a piece cortex of outer table and a great deal of cancellous bone of the ilium were removed in young children, the created bone defects of the ilium could be repaired by active periosteum regenerates without significant complications.^[28]^

In comparison with traditional onlay bone graft, we developed wrapping autogenous cortical and cancellous bone grafts. The former can also provide vital osteoblasts with both of osteoinductive glycoprotein and BMP, whereas the cylindrical critical bone graft, particularly characterized by modality of wrapping cancellous bone have osteoconductive effects.^[29]^ At the same time, this manner of graft contribute to keep the cancellous pieces compacted contact with previous pseudarthrosis and to form sealing environment in location, allowing production of high concentration of glycoprotein and bone marphogenic protein which improve biologic environment and may be key factor for facilitating union of CTP.

In initial radiographs examination after the operations, there was significantly spindle-shaped expansion at the site of wrapping bong graft from our series of 51 patients with CTP, all of wrapping graft sites had no obvious radiolucent between grafts and tibia on both anteroposterior and lateral radiographs, indicating the grafts successful incorporated into recipient site. The spindle-shaped expansion of wrapping graft of tibia had gradually reduced its diameter and possessed sufficient thickness between the both ends of previous pseudarthrosis which is hallmark of remoulding bone grafts at over 3 months following the operation, therefore union criteria established by Ohnishi had been achieved. However, the specific mechanism still lacks clarity and the long-term stability is hard to predict, therefore further researches are required in the future.
